# Widefield imaging of retinal and choroidal tumors

**DOI:** 10.1186/s40942-019-0196-5

**Published:** 2019-12-12

**Authors:** Natalia F. Callaway, Prithvi Mruthyunjaya

**Affiliations:** Department of Ophthalmology, Stanford University Byers Eye Institute, 2452 Watson Court MC 5353, Palo Alto, CA 94303 USA

**Keywords:** Eye, Tumor, Choroid, Retina, Retinoblastoma, Hemangioma, Nevus, Melanoma, Metastasis, Wide-field imaging, Wide-field angiography, wide-field photography, confocal scanning laser ophthalmoscopy, Optos, Heidelberg Spectralis, RetCam 3, Clarus 500, Phoenix Icon, Panoret-1000

## Abstract

**Background:**

Wide-field imaging plays an increasingly important role in ocular oncology clinics. The purpose of this review is to describe the commonly used wide-field imaging devices and review conditions seen in ocular oncology clinic that underwent wide-field imaging as part of the multimodal evaluation.

**Summary of review:**

Wide-field or wide-angle imaging is defined as greater than 50° field of view. Modern devices can reach far beyond this reporting fields of view up to 267°, when utilizing montage features, with increasingly impressive resolution. Wide-field imaging modalities include fundus photography, fluorescein angiography (FA), fundus autofluorescence (FAF), indocyanine angiography (ICG), spectral domain optical coherence tomography (SD-OCT), and recently wide-field OCT Angiography (OCTA). These imaging modalities are increasingly prevalent in practice. The wide-field systems include laser, optical, and lens based systems that are contact or non-contact lens systems each with its own benefits and drawbacks. The purpose of this review is to discuss commonly used wide-field imaging modalities for retinal and choroidal tumors and demonstrate the use of various widefield imaging modalities in select ocular oncology cases.

**Conclusions:**

Clinical examination remains the gold standard for the evaluation of choroidal and retinal tumors. Wide-field imaging plays an important role in ocular oncology for initial documentation, surgical planning, determining the relationship of the tumor to adjacent ocular structures, following tumor size after treatment, and monitoring for recurrence.

## Background

Clinical examination is the gold standard for the diagnosis of retinal and choroidal tumors that often involve pathology in the peripheral fundus. Historically, imaging technology could easily and clearly capture photographs of lesions located in the posterior pole to the edge of the equator. The first fundus camera had a 20° field of view, later expanded to 30°, and this was set as the standard for fundus photography. Over the last few decades advances in imaging modalities have vastly expanded the field of view photographed and enabled microscopic evaluation of the peripheral retina. Wide-field or wide-angle imaging is defined as greater than 50° field of view with modern devices that can reach far beyond this reporting fields of view up to 267° when utilizing montage images. Montage imaging requires photographic expertise to capture and align the images and often fails to obtain far peripheral views in focus with the posterior pole images. Wide-field imaging has found a particularly important role in ocular oncology for initial documentation, surgical planning, determining the relationship of the tumor to adjacent ocular structures, following tumor size after treatment, and monitoring for recurrence.

**Fig. 1:**
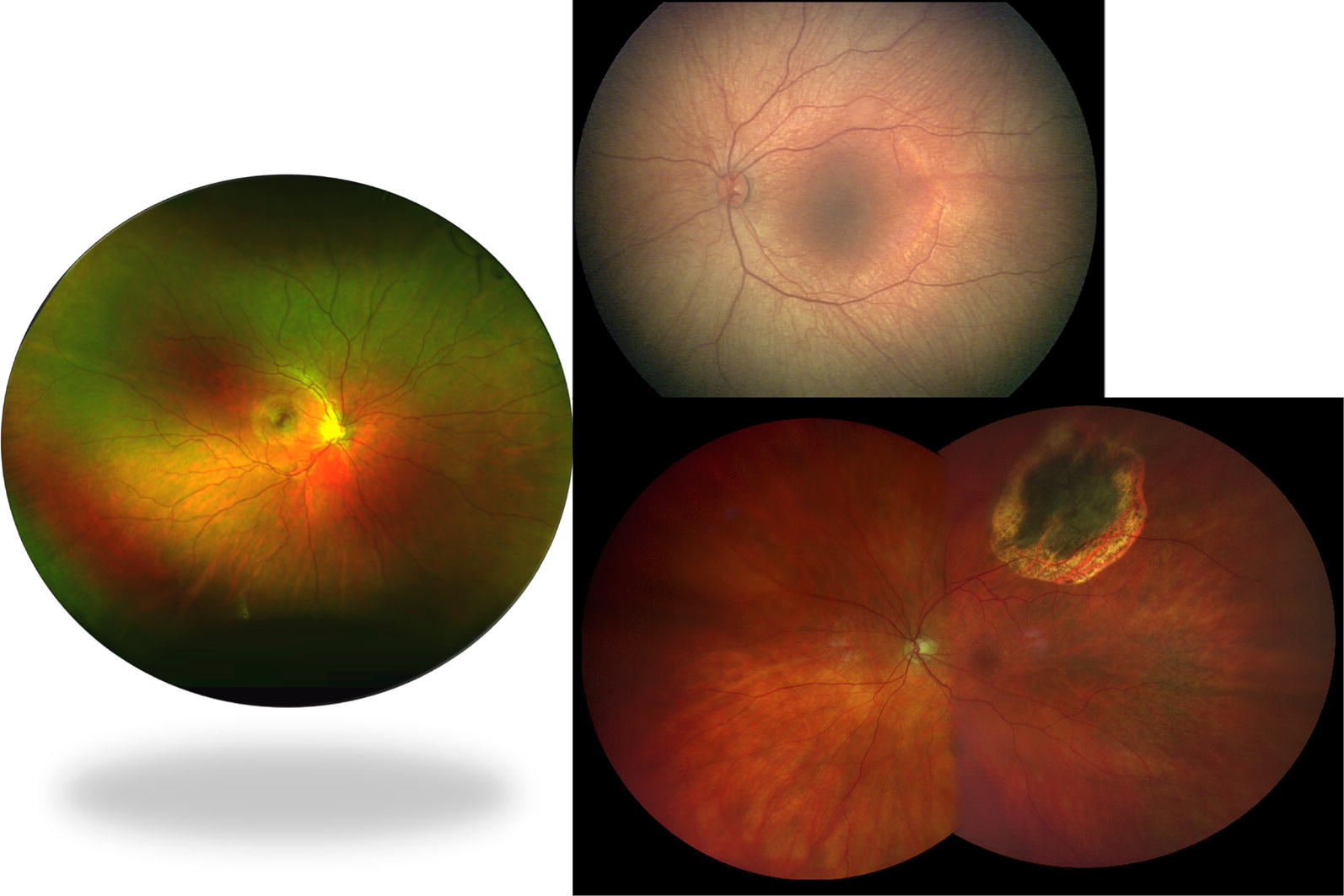
Field of view with various widefield imaging systems. Left: Optos camera (Optos PLC, Dunfermline, UK) offers up to 200° of horizontal field of view. Right Upper: 130° RetCam 3 (Clarity Medical Systems, Inc., Pleasanton, CA, USA). Right Lower: Clarus 500 (Carl Zeiss Meditech Inc., Dublin, CA)

The wide-field systems include laser, optical, and lens based systems that are contact or non-contact lens systems each with its own benefits and drawbacks (Table 1). Commonly used non-contact laser-based systems include Optos (Optos PLC, Dunfermline, UK) and Heidelberg Spectralis (Heidelberg, Engineering, Inc., Heidlberg, Germany). The main limitation of Optos at the time of this review is color distortion due to the green filter over the image. Optos has gained popularity in clinics because it is a non-mydriatic clinic based system with ultra-wide fields of view. The Clarus 500 (Carl Zeiss Meditech Inc., Dublin, CA) was recently released for commercial use and reports the widest field of view with minimal color distortion. Like all wide-field imaging technologies the camera is attempting to capture a three-dimensional structure with a two-dimensional image and this can result in artifacts and peripheral aberrations. Ret Cam 3 (Clarity Medical Systems, Pleasanton, CA) is a contact lens optical system that is commonly used in pediatric ophthalmology because of its wide-field of view and FA capability. Recently, the Phoenix Icon (Phoenix Technology Group, Pleasanton, CA) wide-field contact lens system was released and reports 100° of view and like the RetCam and Optos offers FA. The Heidelberg Spectralis in conjunction with the Ocular Straurenghi 230 SLO Retina Contact Lens (Ocular Instruments, Inc., Bellevue, WA) offers 150° field of view. The contact lens, however, requires patient cooperation and photographer skill to truly capture these wide-angle images. Finally, a contact lens based imaging technology that works via a transscleral as opposed to transpupillary illumination is the Panoret-1000 (CMT Medical Technologies Inc, Valley Stream, NY). Compared to the RetCam 3, the Panoret-1000 can obtain a similar field of view, but better imaging through medial opacities such as corneal edema or cataract. At the time of this review the Panoret-1000 does not have FA or ICG capabilities. Figure 1 demonstrates the field of view obtained with Optos (left) and RetCam 3 (right upper) and Clarus (right lower) wide-field imaging systems.

**Table 1:** Characteristics and capabilities of common wide-field imaging devices

Device	Field of view (up to degrees)	Lens	System	Capabilities
Optos	180–200	Noncontact	Laser	FA, FAF, ICG
Heidelberg Spectralis	55	Noncontact	Laser	FA, FAF, ICG
	150 with lens	Contact	Optical	
RetCam 3	30–130	Contact	Optical	FA
Clarus 500	267	Noncontact	Optical	FAF
Phoenix Icon	100	Contact	Optical	FA

*FA* fluorescein angiography, *FAF* fundus autofluorescence, *ICG* indocyanine angiography

**Fig. 2:**
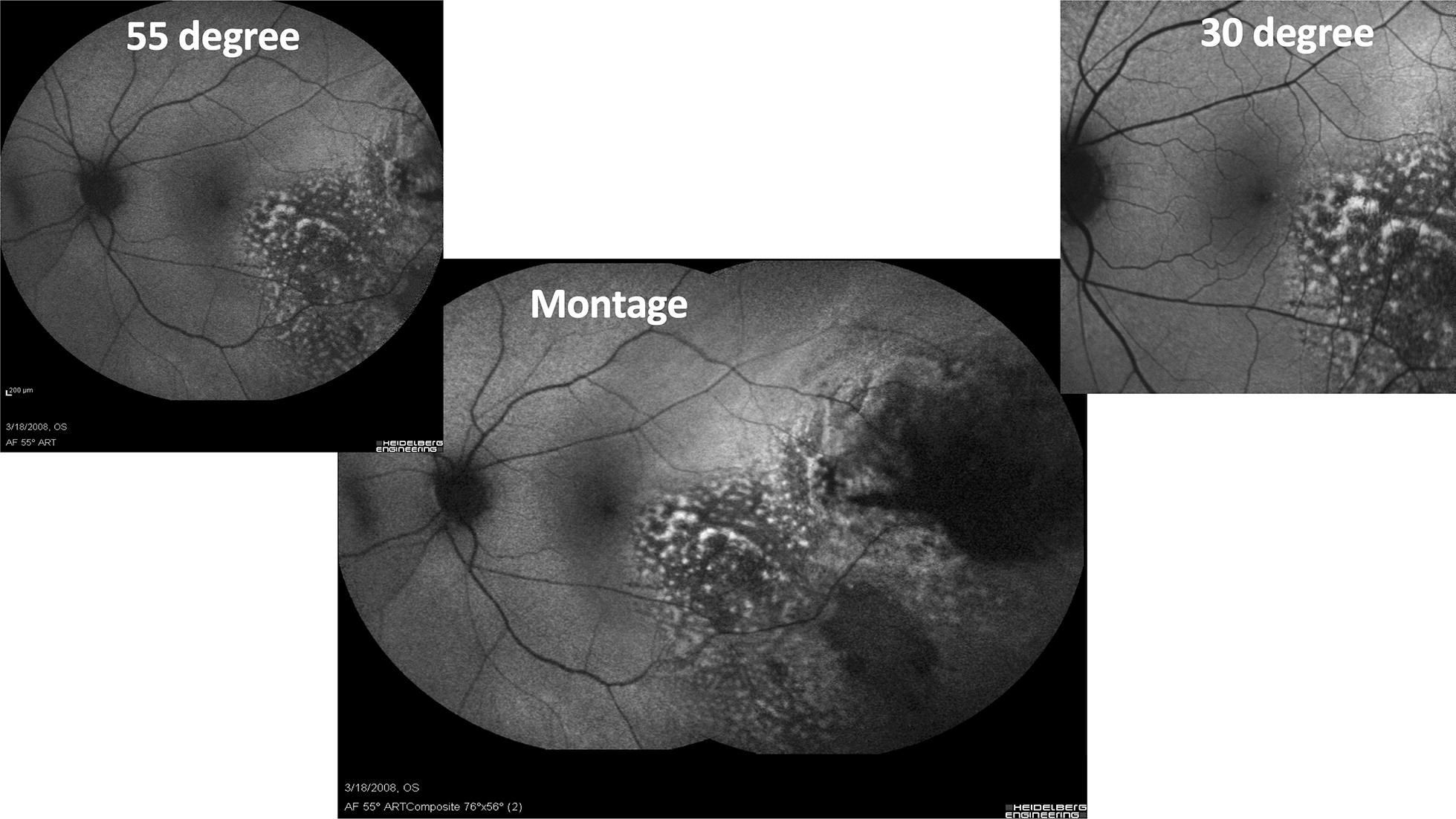
Fundus autofluorescence of a choroidal melanoma with the Heidelberg Spectralis (Heidelberg, Engineering, Inc., Heidlberg, Germany) using a standard 30° image (upper right), an extended 55° image (upper left), and a montage image (center). The choroidal melanoma demonstrates increase hyperautofluorescense corresponding to clinical areas of orange pigment as well as RPE changes

As imaging characteristics of fundus tumors have been better characterized, using multiple imaging modalities may enhance the diagnostic accuracy of the clinician [1]. Wide-field imaging modalities including fundus photography, fluorescein angiography (FA), fundus autofluorescence (FAF) as shown in Fig. 2, indocyanine angiography (ICG), spectral domain and swept source optical coherence tomography (SD-OCT), and recently, wide-field OCT Angiography (OCTA) are increasingly prevalent in practice. Wide-field imaging, often in conjunction with other imaging modalities, allows the clinician to identify and document the baseline lesion characteristics including location, dimension, color, and surface features. The enhanced field of view offered by wide-field imaging assists in recognition and follow-up of lesions with peripheral retinal pathology [2]. Wide-field angiography can be particularly helpful in evaluation of vascular tumors. In a montage of FA, the various images will not be in the same time phase of the angiogram making subtle angiographic changes harder to interpret. FAF is used in ocular oncology to detect lipofuscin, RPE changes, and subretinal fluid that are published risk factors for increased malignant potential when evaluating a melanocytic lesion [3].

After diagnosis, wide-field imaging can assist with surgical planning by documenting lesion margins and evaluate the response to treatment with serial images to quantify interval changes. It can also identify the extent of retinal non-perfusion, radiation retinopathy, and other tumor effects that may not be readily apparent on clinical examination.

The purpose of this review is to discuss the most commonly used wide-field imaging modalities for retinal and choroidal tumors and demonstrate the use of various widefield imaging modalities in select ocular oncology cases.

### Retinoblastoma

**Fig. 3:**
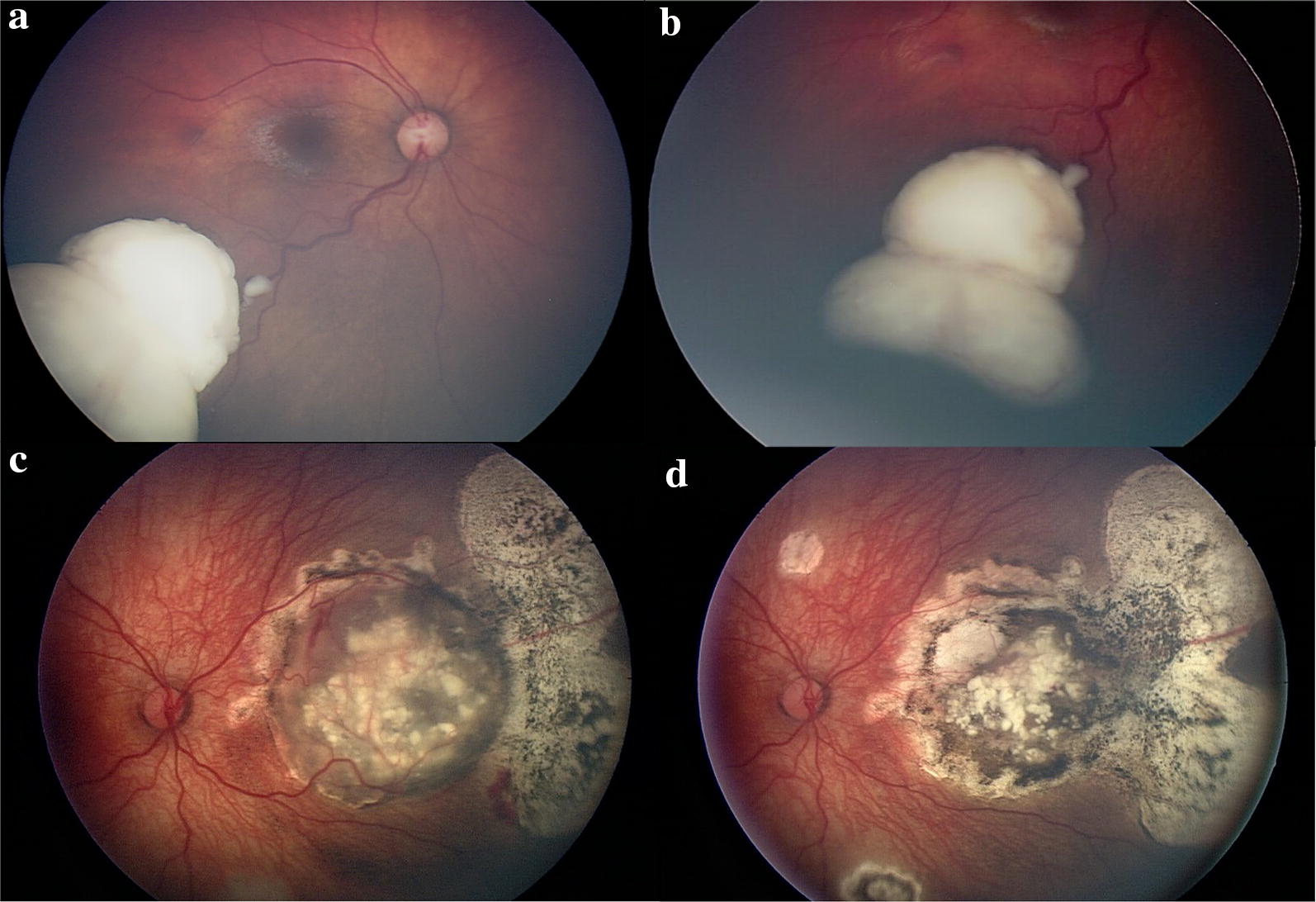
Retinoblastoma imaging with RetCam 3 (Clarity Medical Systems, Inc., Pleasanton, CA, USA). **a** Retinoblastoma along the inferotemporal arcade with dilated feeding and draining retinal vessels. **b** Retinoblastoma anterior margin captured with scleral depression. **c** Large nasal retinoblastoma with calcifications and chorioretinal changes in the periphery. **d** Shows regression of the tumor following treatment

Retinoblastoma is the most common intraocular malignancy among children. Most often it presents with leukocoria, strabismus, or ocular inflammation and requires prompt examination under anesthesia and treatment once the diagnosis is confirmed. Retinoblastoma can have a wide range of clinical presentations but is classically a gray-white intraretinal mass fed and drained by retinal vessels and can have various associated ocular findings based on the affected structures Fig. 3. The tumor is bilateral in 30–40% of patients.

Wide-field imaging offers a reliable and quantitative way to monitor response to treatment. Contact lens systems, such as the RetCam 3, are typically used for retinoblastoma evaluation and imaging because non-contact images are difficult to obtain on these young patients in clinic. The RetCam has a more limited field of view, but increased depth compared to Optos. Scleral depression can aid in obtaining the full retinal periphery in the RetCam field of view (Fig. 3b). Changing the focus plane to the anterior segment or vitreous can detect anterior changes and vitreous seeding that are critical in retinoblastoma classification. Kim et al. found RetCam FA to assist in the diagnosis and initial description of retinoblastoma by highlighting features more common with this malignancy including subclinical iris neovascularization, retinal vessel abnormalities, retinal venous leakage, and intrinsic tumor vessels [4]. Wide-angle OCT has been reported to assist in the diagnosis of retinoblastoma in indeterminate cases [5, 6].

### Angiomatous tumors

The differential for an angiomatous tumor in the choroid or retina includes retinal capillary hemangioblastoma, retinal cavernous hemangiomas, arteriovenous (AV) malformations, and choroidal hemangiomas. Wide-field imaging particularly FA and ICG offer key diagnostic information for these lesions.

#### Retinal capillary hemangioblastoma

**Fig. 4:**
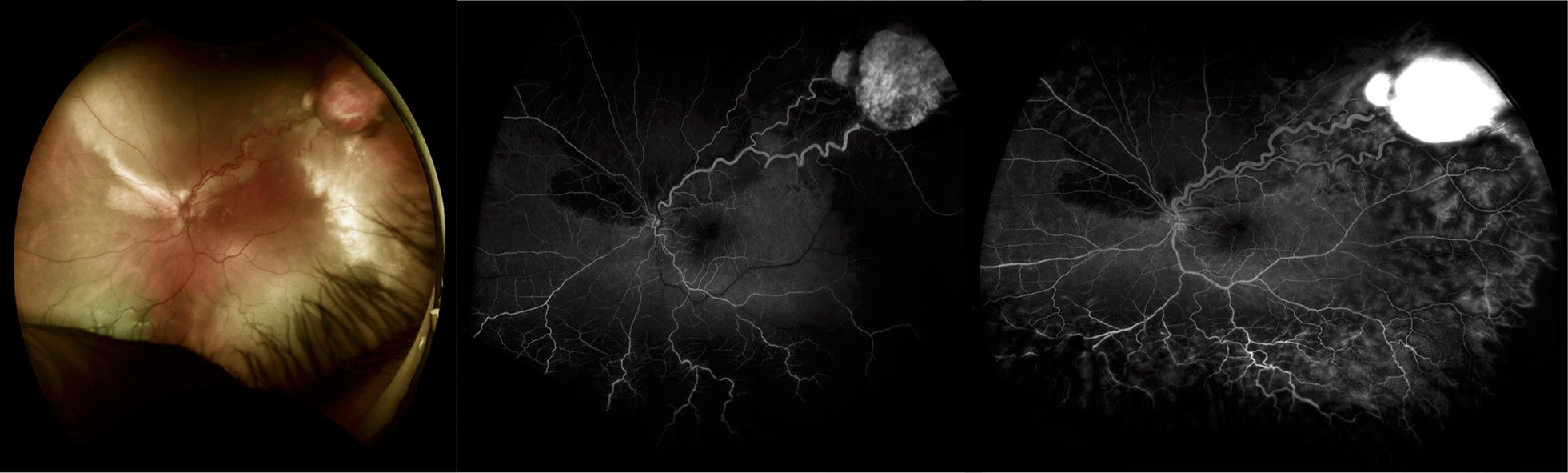
Optos wide-field fundus photo (left) and FA (middle and right images) of a temporal VHL associated retinal hemangioblastoma in the left eye. Wide-field imaging and FA play an important role as the lesions are often peripheral and demonstrate a classic FA pattern of rapid filling of the feeding retinal arteriole, later filling of the intralesional blood vessels, followed by drainage through he dilated venule Left Optos wide-field image. Middle: Early phase (31 s) at arteriolar filling demonstrating rapid dye. Right: Later phase (1:02 min) with rapid dye filling into the hemangioblastoma and leakage of the dye

Retinal capillary hemangioblastomas are a rare angiomatous tumor arising from the retina that presents as a reddish-orange mass with large, dilated feeding and draining retinal vessels. Wide-field imaging and FA play an important role as the lesions are often peripheral and demonstrate a classic FA pattern of rapid filling of the feeding retinal arteriole, later filling of the intralesional blood vessels, followed by drainage through the dilated venule and massive late leakage into the tumor and vitreous (Fig. 4). In one study, wide-field imaging detected more hemangioblastomas than ophthalmoscopy and standard angiography because of the often far peripheral location of these lesions [7]. In isolation the condition is termed *von Hippel disease*, however, if it is associated with cerebellar hemangioblastomas or other systemic symptoms it is termed *von Hippel*-*Lindau syndrome.* Referral for genetic testing, neurologic, nephrological, and systemic evaluation is critical as patients are at increased risk for cerebellar hemangioblastomas, renal cell carcinoma, and pheochromocytoma. Regular ophthalmic evaluation and early treatment of hemangioblastomas can reduce the late complications of this condition including exudative retinal detachment.

#### Retinal cavernous hemangioma

FA is nearly diagnostic of retinal cavernous hemangiomas that are a rare retinal tumor that clinically presents with a “cluster of grapes” appearance. The tumor consists of dilated, thin-walled vascular channels and within these plasma-erythrocyte separation occurs. As a result, the FA reveals a slowly filling lesion with fluorescein that pools at the top of the space (within the plasma segment) and remains there for a prolonged period of time without leakage of the dye. Treatment of these lesions is rarely required, however, referral for systemic evaluation for dermatologic and neurologic manifestations is warranted.

#### Retinal arteriovenous malformation

**Fig. 5:**
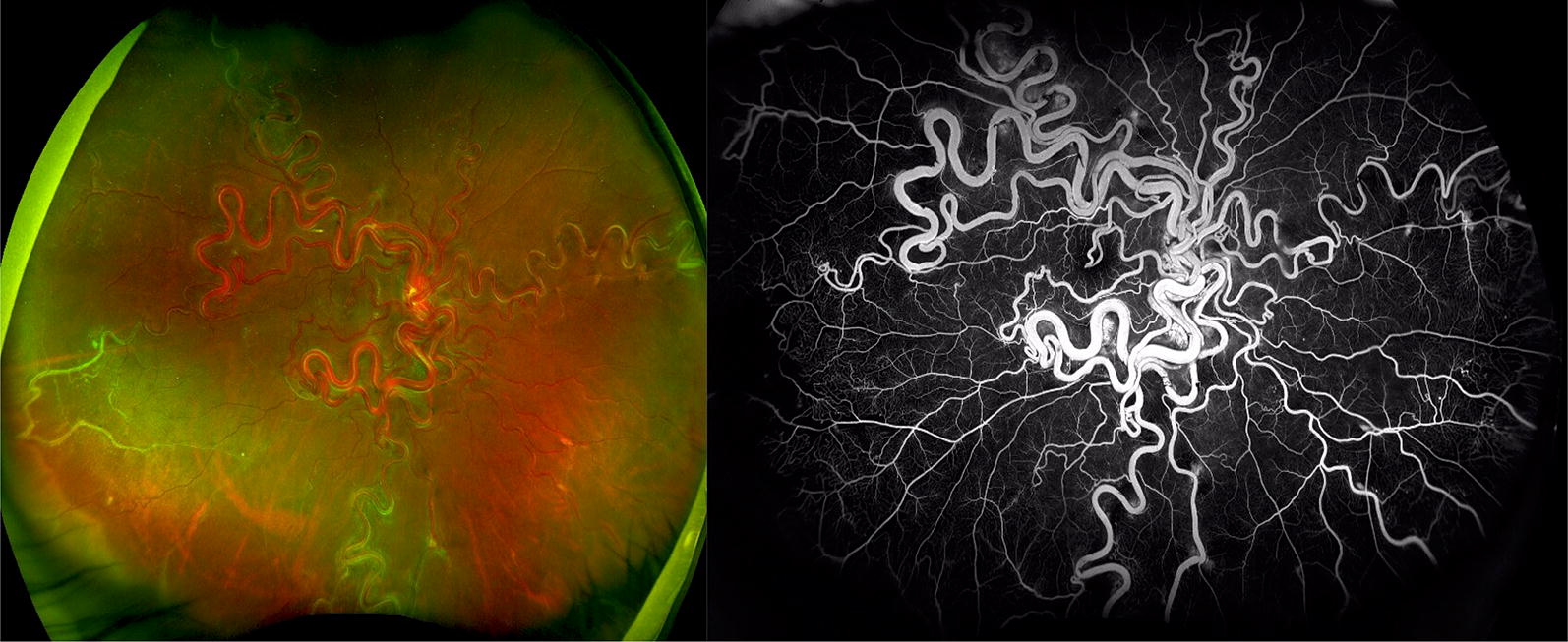
Wyburn-Mason Racemose Hemangioma Optos Wide-field Fundus Imaging on the left and Fluorescein Angiography on the right. Fundus photo demonstrates a large racemose hemangioma with a temporal sclerotic vessel. The fluorescein angiography reveals temporal non-perfusion. These images were originally published in the Retina Image Bank^®^ website. Authors: Sarina Amin and Neal Palejwala. Photographer: Sarah Ellano. Title: Wyburn-Mason Widefield Fundus Photography and Fluorescein Angiography. Retina Image Bank. April 29 2018. © the American Society of Retina Specialists. Images 28152 and 28153

Also known as a racemose hemangioma (Fig. 5) represents an anomalous anastomosis between the arterial and venous system in the retina that can be associated with intracranial AV malformations constituting *Wyburn*-*Mason syndrome.* FA demonstrates characteristic rapid filling of both the afferent and efferent retinal vessels without an intervening capillary bed and no leakage of the dye.

#### Choroidal hemangioma

There are two distinct forms of choroidal hemangiomas: circumscribed and diffuse. Circumscribed lesions are benign, reddish-orange tumors with high reflectivity on ultrasound and do not have systemic associations. These are typically located within the posterior pole and are captured well with traditional fundus imaging. Diffuse tumors present with a saturated red appearing fundus referred to as *tomato ketchup fundus* and is associated with encephalofacial angiomatosis (Sturge-Weber syndrome). FA highlights the large choroidal vessels in these lesions in the early phases with late staining of the tumor. Wide angle fundus photography can help monitor the degree and resolution of associated exudative retinal detachments. Sturge-Weber patients require dermatologic and neurologic evaluation for other potentially life-threatening hemangiomas.

#### Retinal vasoproliferative tumors

These lesions represent a benign vascular mass in the retina often found in otherwise healthy individuals. The lesions can cause damage through direct mass effect, or adjacent exudation and resulting macular edema. They often have borders that extend into the mid-periphery that can be visualized with wide-field imaging and FA.

### Melanocytic lesions

The differential for a melanocytic choroidal lesion include choroidal nevus, melanoma, congenital hypertrophy of the retinal pigment epithelium (CHRPE), melanocytoma, adenomas and adenocarcinomas of the nonpigmented and pigmented ciliary epithelium, combined hamartoma of the RPE and retina, metastasis, atypical retinal or choroidal hemorrhage, hemangiomas. Wide-field imaging particularly FA in conjunction with examination, ultrasound, and if necessary tissue biopsy or CT/MRI can assist in determining the correct diagnosis and treatment plan.

When evaluating melanocytic lesions, laser projection systems can leave shadows from any anterior opacity including focal cataracts, vitreous opacities, or the lashes or nose, that give the appearance of pigmented lesions or pseudomelanocytic lesions [8]. Furthermore, some of the laser imaging technologies may not always represent true color, which is an important characteristics in the evaluation of a melanocytic lesion. In these cases a standard true color photograph may be required.

#### Choroidal nevus

Choroidal nevi are incidentally detected in 6–10% of subjects and represent a relatively common ophthalmic condition detected on routine examination [9, 10]. Nevi classically are an asymptomatic, flat or minimally elevated, pigmented choroidal lesion. Rarely they can be amelanotic. The initial evaluation of a pigmented choroidal melanocytic lesion is aimed at determining if the lesion is malignant.

Once a lesion has been determined to be a choroidal nevus, regular follow-up and photographic documentation is recommended at baseline and follow-up. Wide-field photographs can capture the full margins of a nevus in the periphery as well as its relationship to other structures such as the retinal vessels to facilitate future comparisons [11]. Although wide-field imaging such as the Optos can capture the size and location of peripheral nevi, the filter can alter the specific color features of nevi. One review recommended capturing both the optic disc and macula in the same image as the nevus to create a baseline comparison color. If this is not possible than a standard fundus photo with true color may be additionally beneficial [12]. Choroidal nevi confer a 1 in 8000 risk of transformation into malignant melanoma and a lifetime risk of growth of approximately 1%. Ocular and oculodermal melanocytosis may predispose to uveal malignancy and require regular examinations because of the lifetime risk of 1 in 400 among Caucasians.

### CHRPE

CHRPE is a sharply demarcated, flat, typically darkly pigmented lesion often with lacunae in young adults. Optomap imaging in the optometric population reported a 1.2% prevalence of CHRPE lesions most commonly in the far temporal peripheral retina [13]. The lesion is asymptomatic and benign in isolation, but multiple patches, particularly at a younger age may herald Gardner syndrome, of familial polyposis, which carries and increased risk of colon carcinoma. Referral to gastroentereology and genetics for the patient and family members is warranted. Wide-field FA of CHRPE with lacunae demonstrates loss of pigmentation and RPE architecture and visible choroidal vessels.

#### Melanoma

Choroidal melanomas are the most common primary intraocular malignancy in adults. They typically present as large, pigmented, elevated, dome-shaped lesions that may be associated with orange pigment, subretinal fluid, RPE changes, or a serous retinal detachment. Orange pigment is due to the contrast of lipofuscin and melanin and is associated with malignant activity. Wide-field FAF enables detailed view of orange pigment and comparison over time (Fig. 2).

**Fig. 6:**
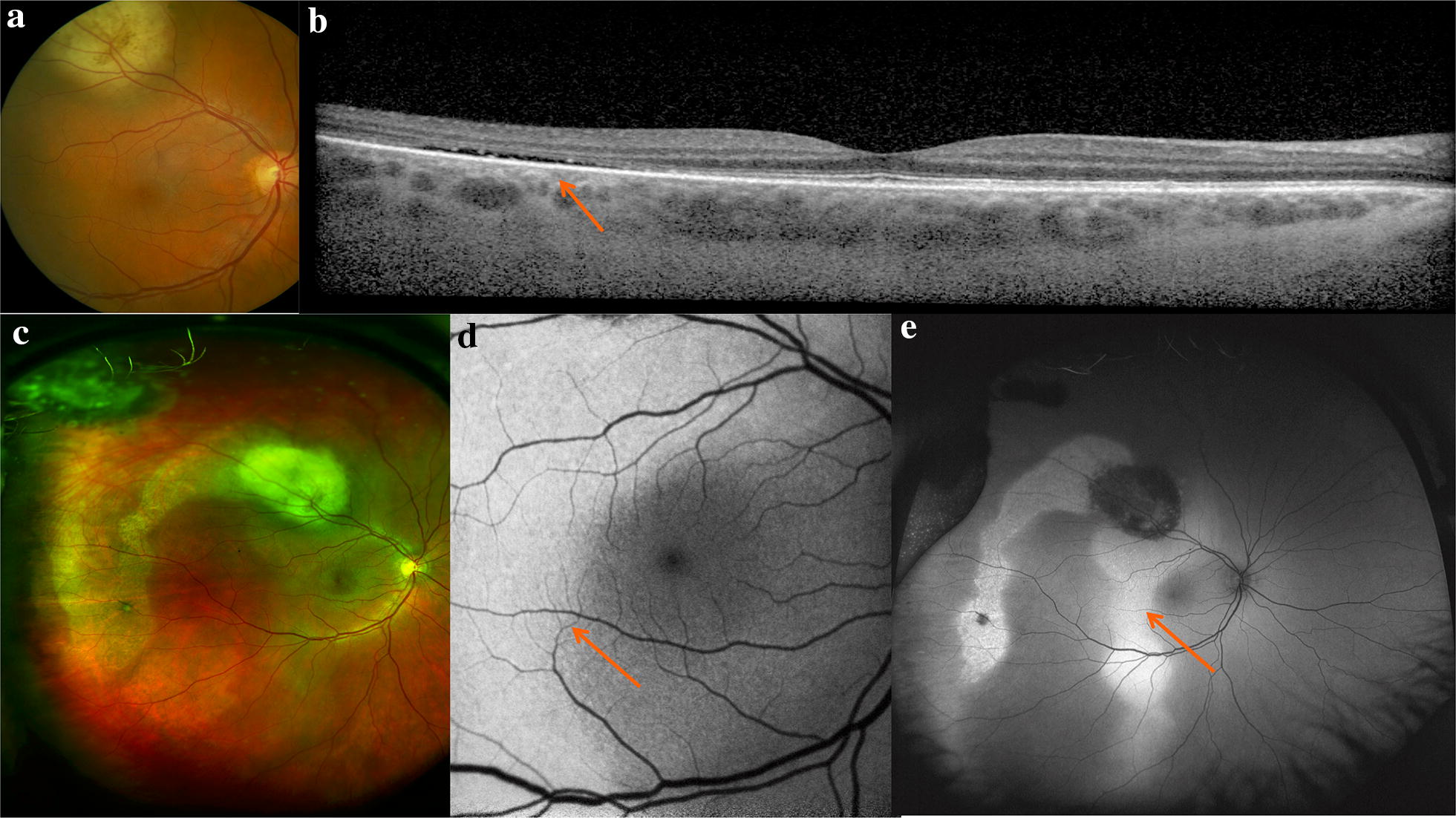
Multimodal imaging in the diagnosis of a uveal melanoma. The patient presented with symptoms of vertical ribbons in his vision and was referred to oncology after fundus examination revealed an elevated lesion along the superotemporal arcade show in the standard photograph (**a**). OCT through the macula (**b**) demonstrates temporal subretinal fluid (arrow) corresponding to the subretinal fluid and associated pigmentary changes seen on the Optos wide-field images (**c**). Fundus autofluorescence of the macula (**d**) and wide-field with Optos (e) highlight the hyperautofluorescence of the subretinal fluid track.

**Fig. 7:**
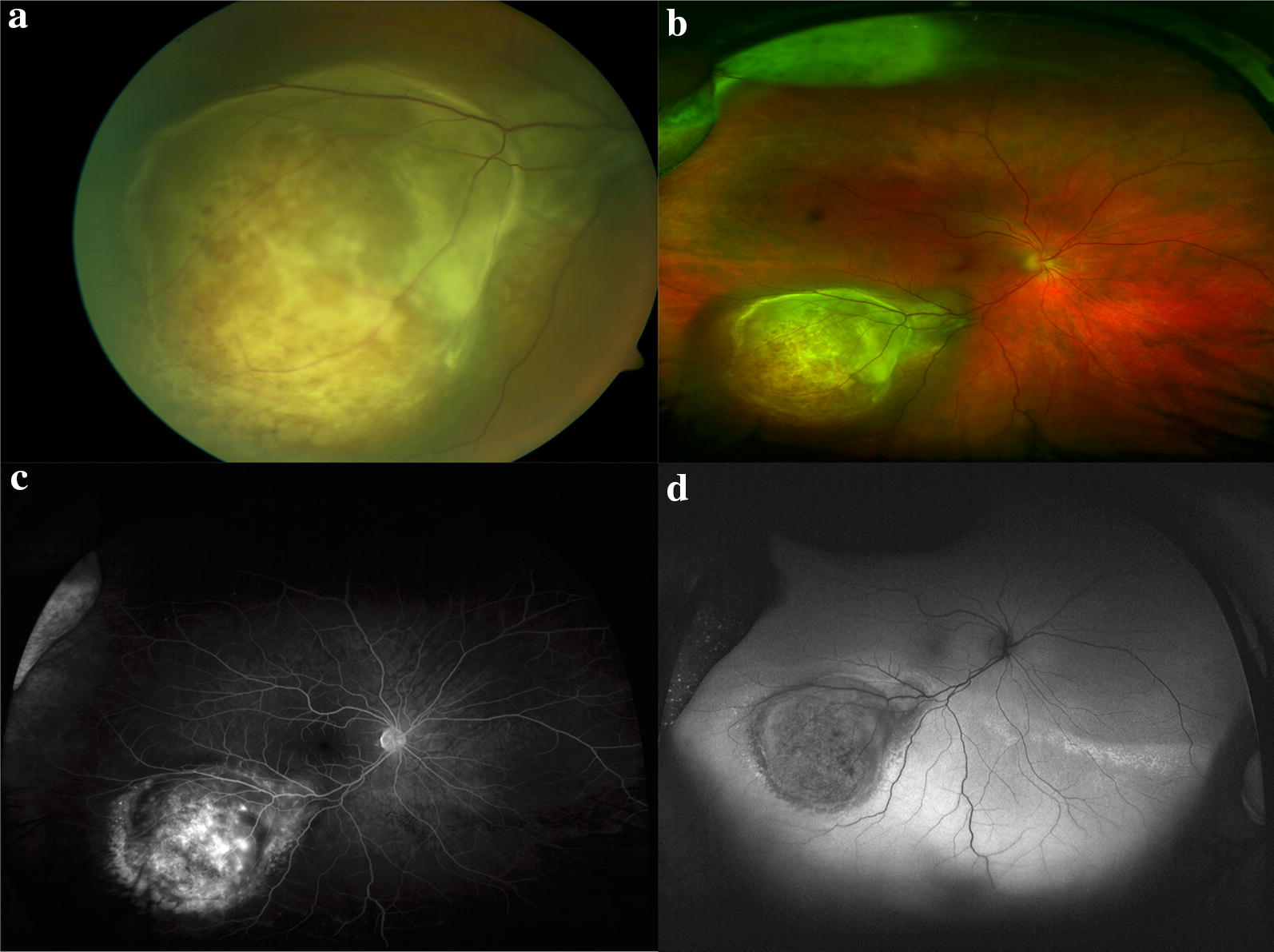
multimodal imaging of uveal melanoma. **a** Standard fundus photography demonstrating a choroidal mass with overlying retinal pigmentary changes and small area of detachment and subretinal fluid. **b** Optos wide-field imaging fo the same mass capturing the peripheral margins of the tumor. **c** Optos fluorescein angiography demonstrates irregular filling of the mass without other lesions visible in the retina. **d** Wide-field fundus autofluorescence of the tumor reveals hypo autofluorescence of the mass with inferior hyperautofluorescence consistent with subretinal fluid on examination

Clinical examination, multimodal imaging, tissue biopsy and systemic evaluation are critical in suspected uveal melanomas (Figs. 6 and 7). Wide-field imaging is used to monitor response to treatment and follow associated radiation retinopathy (see below).

Wide-field imaging offers the advantage of imaging tumors in the periphery [14] without requiring montage imaging. Standard cameras cannot focus on anteriorly placed lesions and are often unable to image the borders of more peripheral lesions. One study reported 90% sensitivity and 82% specificity of clinical examination and Optomap ultra-wide field imaging in differentiating between a choroidal nevus and a melanoma [9]. A different study reported high specificity (85%) and moderate sensitivity (74%) for detection of lesions posterior to the equator, but low sensitivity (45%) for lesions anterior to the equator [15].

Wide-angle imaging of melanomas during initial diagnosis documents the initial size of the lesion and its relationship to adjacent structure to monitor response to therapy. Wide-field photography has been reported to yield similar tumor size measurements as ultrasonography when the tumor is less than 3 mm in height and there is no associated subretinal fluid or detachment [16]. There are no pathognomonic FA findings for choroidal melanoma, however, prominent early choroidal filling and a double circulation pattern has been reported. In one study, wide-angle Optos FA facilitated the diagnosis of choroidal melanoma in dense asteroid hyalosis [17]. Other group found that among patients with known choroidal melanoma, nonmydriatic scanning laser ophthalmoscopy with Optomap and clinical examination resulted in a 90% sensitivity and 82% specificity in differentiating malignant from nonmalignant lesions. This study requires validation with future investigation [9].

### Metastasis

**Fig. 8:**
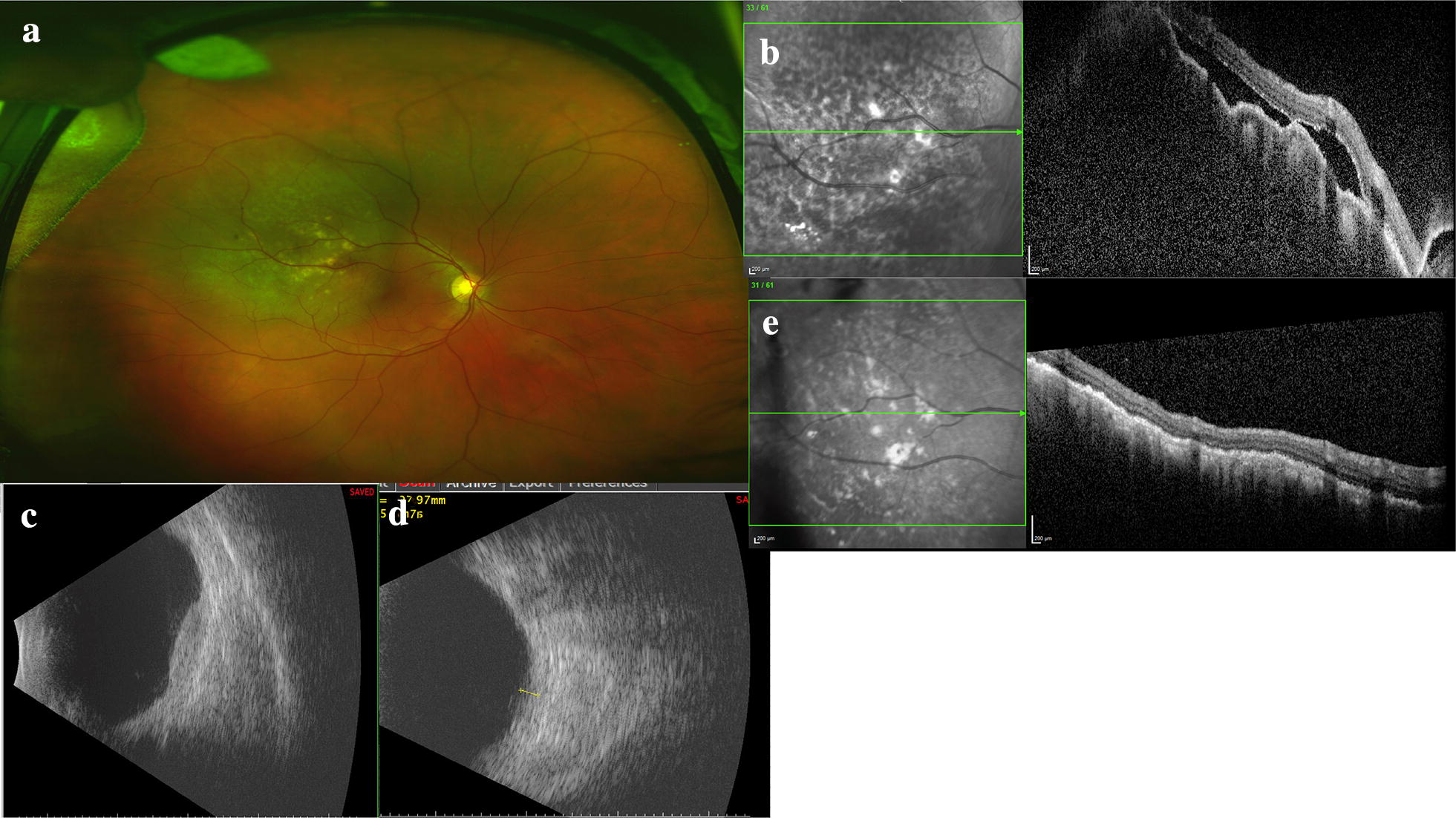
Multimodal imaging of a uveal metastasis from lung adenocarcinoma. The patient presented with reduced vision in his right eye and clinical examination revealed a large, placoid-like mass at the superotemporal arcade with overlying pigmentary changes and associated fluid. **a** Optos wide-field imaging of the placoid lesion with overlying pigmentary changes. **b** OCT with en face image with pigmentary changes, irregular, low-lying choroidal mass and resulting undulations of the choroid and associated subretinal fluid. **c** Ultrasonography reveals the lesion is irregular. **d** After target treatment the lesion thickness decreases on ultrasonography and **e** the choroidal mass has resolved and the subretinal fluid absorbed

The most common intraocular and intraorbital tumor in adults is metastatic disease and thus the recognition of such lesions is critical. The choroid is frequently the site of involvement because metastatic foci spread hematogenously from distant organs. The most common primary site of the cancer varies by gender. Females most often present with breast carcinoma followed by lung carcinoma while males most often present with lung carcinoma [18]. Wide-angle imaging is helpful in baseline imaging of both eyes to capture all lesions and FA aids in defining the margins of the metastatic tumor [19]. The metastatic lesion often alters the overlying RPE and results in FAF changes as well as pigmentation patterns such as “leopard spotting”. Wide-field imaging in conjunction with other modalities can also be used to monitor response to treatment and serially for evidence of recurrence (Fig. 8).

### Radiation retinopathy

**Fig. 9:**
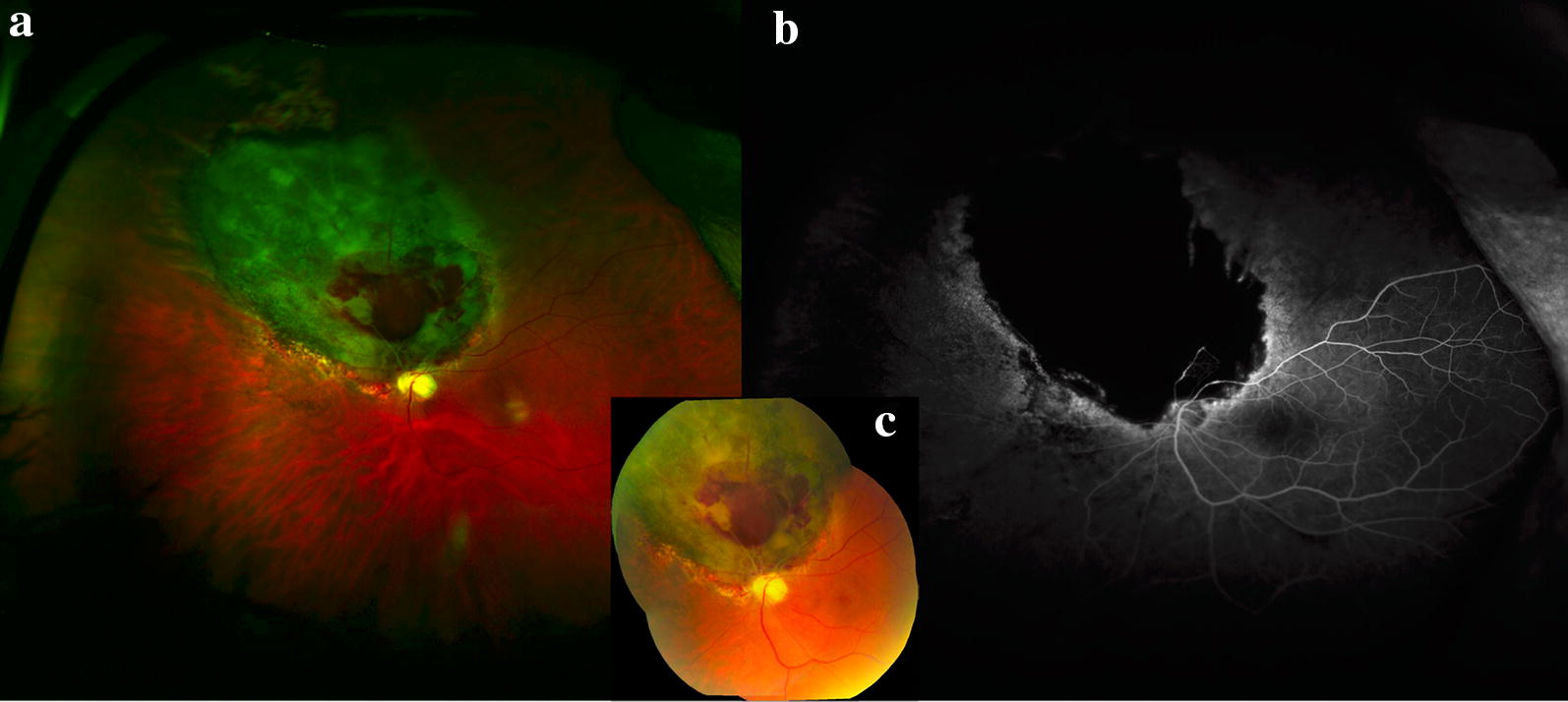
Radiation retinopathy following melanoma treatment. **a** Optos wide-field image of radiation retinopathy following melanoma treatment with brachytherapy. Superonasal to the disc is a flat chorioretinal scar with retinal hemorrhage and adjacent pigmentary changes. **b** Wide-field Optos fluorescein angiography reveals nonperfusion in the area of the scar with late leakage at the border with early development of radiation retinopathy. **c** Montage fundus photo demonstrating superonasal hemorrhage and radiation retinopathy

Radiation retinopathy is a dose-dependent complication of radiation exposure with presentation similar to diabetic retinopathy (Fig. 9). Complications from radiation retinopathy can result in irreversible vision loss and may require intervention with laser photocoagulation, intravitreal injection, or vitreoretinal surgery to reduce visual morbidity. Wide-field imaging, particularly wide-field FA can aid in the staging of radiation retinopathy by quantitatively assessing peripheral nonperfusion and neovascularization [20].

## Conclusions

Over the last several decades there have been significant advances in retinal imaging technology that have enriched our understanding of various retinal conditions. High resolution images allow for visualization of pigment characteristics, vascular leakage, ellipsoid zone health, details of each retinal layer, presence or absence of the RPE, and recently blood flow and oxygenation, that provide further insight into choroidal and retinal tumors. Modern devices have rapid image acquisition times, short processing times, and offer transmission of photography data to other units or the electronic health record. Wide-field imaging plays an important role in the diagnosis, management, and follow-up of retinal and choroidal tumors as these lesions often extend or present in the periphery or have important vascular changes in the periphery that can be identified and better characterized with wide-field imaging. Machines with multiple modalities of simultaneous, or at least sequential, imaging aid in the evaluation and management of peripheral lesions. For this reason, it is now the standard of care to obtain multimodal imaging of choroidal and retinal tumors in ocular oncology clinic.

The exact utility of each camera and wide-field imaging type remains controversial and further research is required in this field. Technology advances at an exponential rate so shortcomings such as resolution, color correction, and field of view will only continue to improve with time. It is important to recognize that despite their proliferation in ophthalmology, wide-field imaging systems have drawbacks that should be considered when evaluating their utility in clinical practice.

As these technologies develop, the increasing field of view and resolution offer potential new insights into the pathophysiology of these tumors and better delineation of these lesions for surgical planning. In the future it is likely that various forms of wide-field imaging systems including OCTA will play an increasingly important role across the spectrum of retinal disease including ocular oncology and these wide-field systems may play a role in screening select high-risk populations. More research into these areas is required to better understand define utility of these images in the treatment of patients with retinal and choroidal tumors. Despite these exciting advances in technology, the gold standard for evaluation of choroidal and retinal tumors remains the clinicians examination.

## Data Availability

Not applicable.
